# P-1329. Practice Patterns and Clinical Outcomes of Standalone Avibactam with Aztreonam Compared to Ceftazidime-Avibactam with Aztreonam for Carbapenem-Resistant Enterobacteriaceae: A Retrospective Study

**DOI:** 10.1093/ofid/ofaf695.1517

**Published:** 2026-01-11

**Authors:** Krishna S Athmakuri, Venkat R Kola, Swathi Prakasham

**Affiliations:** Yashoda Hospitals, Hitech City, Hyderabad, Telangana, India; Yashoda hospitals, Hyderabad, Telangana, India; Yashoda Hospitals, Hitech City, Hyderabad, Telangana, India

## Abstract

**Background:**

Ceftazidime-Avibactam with Aztreonam has been the standard treatment for New Delhi metallo-β-lactamase(NDM)-producing Carbapenem-Resistant Enterobacteriaceae (CRE) infections. NDM and Ambler class D enzymes predominate in India, in contrast to developed countries where KPC predominates. With the recent availability of standalone Avibactam in India, we aim to compare the practice patterns and clinical effectiveness of Avibactam-Aztreonam (AVI-ATM) versus Ceftazidime-Avibactam with Aztreonam(CZA-AVI-ATM) in CRE infections.

**Figure:**
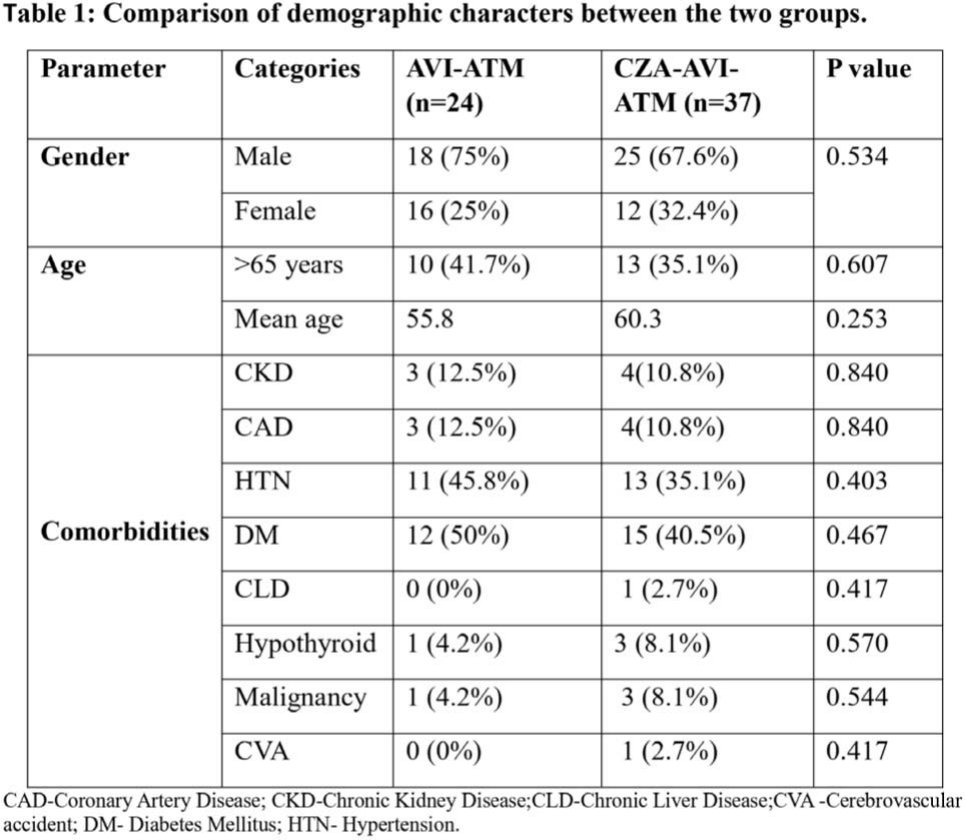
None of the demographic characteristics varied significantly between the two groups.

**Figure:**
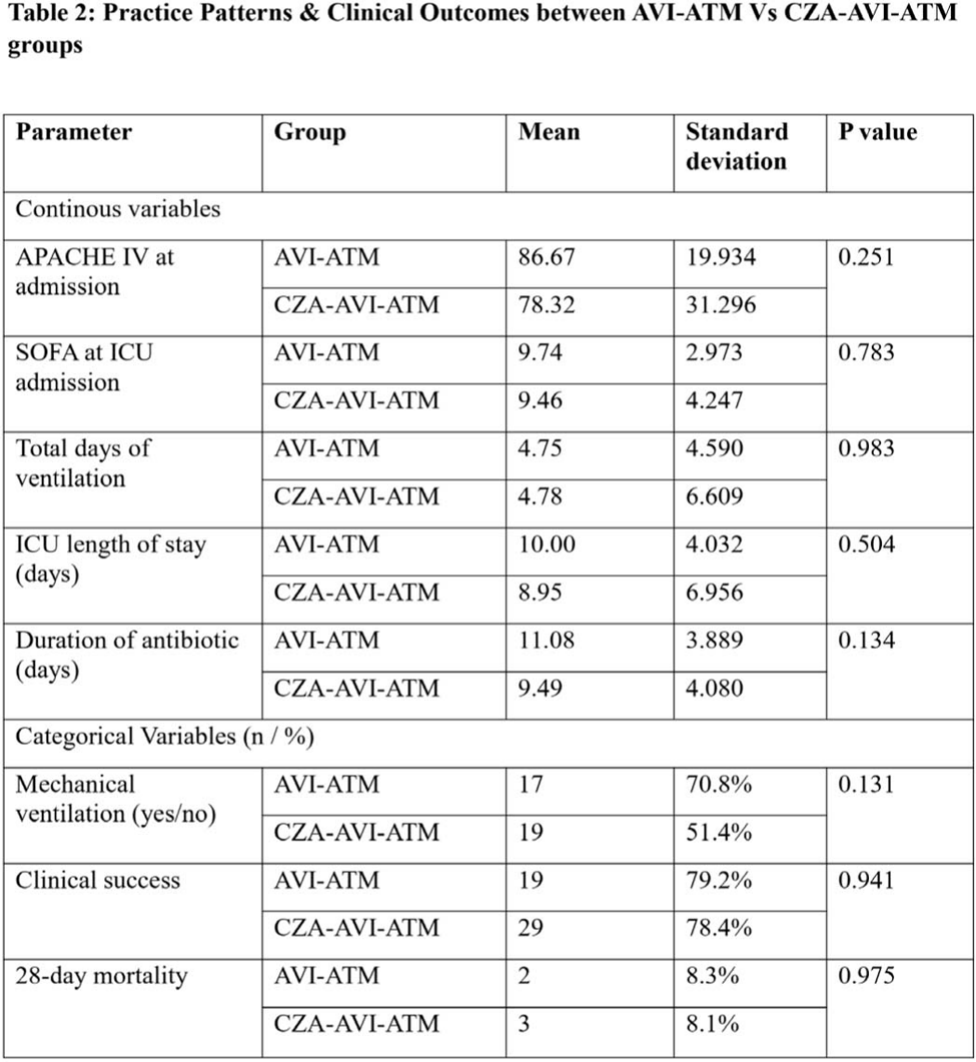
None of the treatment details varied significantly between the two groups

**Methods:**

A single-centre retrospective observational study was conducted in a tertiary care ICU in South India, over 10 months (July 2024 to April 2025). Clinical data were retrieved from the institutional ICU database. Practice patterns and clinical outcomes were compared between patients with culture-confirmed CRE infections treated with AVI-ATM and CZA-AVI-ATM. Resistance genes were identified using the Xpert Carba-R assay.

**Figure:**
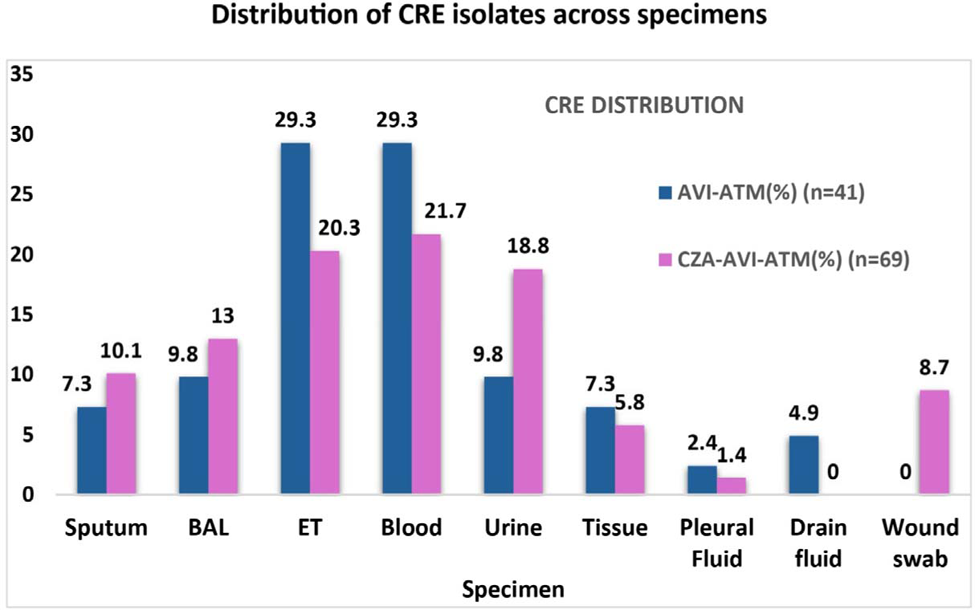
BAL- Bronchoalveolar lavage; ET- Endotracheal aspirate

**Figure:**
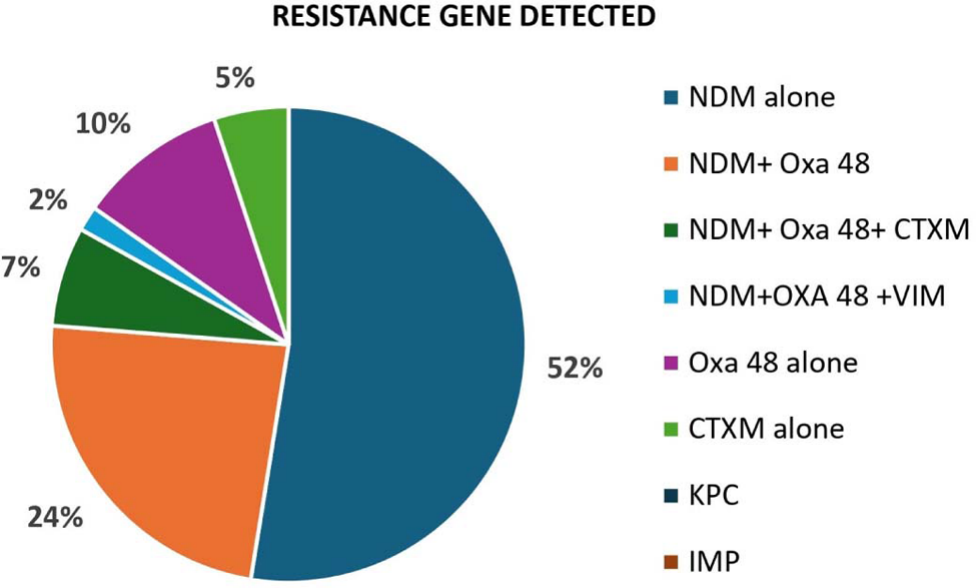
NDM was the most frequently detected resistance gene among all the isolates tested.

**Results:**

A total of 61 patients were included in the study, 24 in the AVI-ATM group and 37 in the CZA-AVI-ATM group. Baseline demographic characteristics, as well as APACHE IV and SOFA scores at admission, did not differ significantly between the groups. Clinical parameters such as duration of antibiotic therapy, need for mechanical ventilation, total ventilator days, length of ICU stay, clinical success (ICU discharge), and 28-day mortality were comparable between the two groups (p > 0.05).

Microbiology: Among 110 culture-confirmed CRE isolates, 24.5% were from blood, 23.6% from tracheal aspirate, 15.5% from urine, 11.8% from bronchoalveolar lavage, sputum 9.1%, tissue 6.4%, wound 5.5%, pleural fluid and drain 1.8% each, and none in cerebrospinal fluid.

Resistance: Carbapenamase gene was identified in 59 of 110 isolates. Among these, 52.54% harbored NDM gene alone, while NDM+OXA-48 gene was in 23.72%, NDM+OXA-48+ CTX-M in 6.78%, NDM+OXA-48 + VIM in 1.69%, OXA-48 gene alone in 10.17%, and CTX-M alone in 5.08%. Overall, NDM was detected in 84.7% of the 59 isolates.

**Conclusion:**

Clinical outcomes were comparable between patients treated with Avibactam with Aztreonam and those who received Ceftazidime-Avibactam with Aztreonam. Notably, the NDM gene was the most frequently identified resistance mechanism in our study.

**Disclosures:**

All Authors: No reported disclosures

